# Association of BMI Change with New-Onset or Progressive Diabetic Kidney Disease in People with Normal-Weight Type 2 Diabetes

**DOI:** 10.3390/jcm15083125

**Published:** 2026-04-20

**Authors:** Lina Mao, Eisha Adnan, Zhuo Chen, Yan Pan, Xiangjun Chen, Tinghua Zan, Shichun Huang, Yujie Wu, Lingjun Sun, Wenyuan Lv, Tingting Luo, Jinbo Hu, Shumin Yang, Qifu Li, Lilin Gong, Zhihong Wang

**Affiliations:** 1 Department of Endocrinology, The First Affiliated Hospital of Chongqing Medical University, Chongqing 400016, China; 15086643092@163.com (L.M.); mahereisha@163.com (E.A.); q1219843728@163.com (Z.C.); cqmu0127@163.com (Y.P.); cqmu1111@163.com (X.C.); cqmu3333@163.com (T.Z.); cqmu6666@163.com (S.H.); 13518488303@163.com (Y.W.); sunlingjun_cu99@163.com (L.S.); cqmu5555@163.com (W.L.); luotingting268@163.com (T.L.); cqmu8888@163.com (J.H.); q443068494@163.com (S.Y.); liqifucqmu@163.com (Q.L.); 2 Department of Endocrinology, The Affiliated Dazu’s Hospital of Chongqing Medical University, Chongqing 402360, China

**Keywords:** type 2 diabetes mellitus, normal weight, BMI change, diabetic kidney disease

## Abstract

**Aims:** This study aimed to examine the association between three-year changes in body mass index (BMI) and the risk of new-onset or progressive diabetic kidney disease (DKD) among people with type 2 diabetes and a normal BMI at baseline. **Methods:** A total of 416 people with type 2 diabetes (T2DM) and a normal BMI were enrolled from the Chongqing Diabetes Registry (CDR, NCT03692884) cohort and were followed for incident DKD until 2025. The change in BMI at the three-year follow-up was classified as follows: stable BMI (<5% change), decreased BMI (≥5% reduction), and increased BMI (≥5% gain). Cox proportional hazards models were used to analyze the association between BMI change categories and DKD risk. **Results:** During a mean follow-up of 3.4 years, people with an increased BMI exhibited a significantly higher risk of DKD onset or progression compared with people with a stable BMI [HR = 1.67, 95%CI: 1.15–2.43, *p* = 0.007]. Each 1% increase in BMI was significantly associated with an increased risk of DKD onset or progression [HR = 1.05, 95%CI: 1.02–1.07, *p* < 0.001]. This association remained significant after multivariable adjustment. Time-dependent receiver operating characteristic (ROC) curves showed that the area under the curve (AUC) of this indicator reached 0.683–0.729 for the prediction of new-onset or progressive DKD risk over 3–5 years. In subgroup analyses, decreased BMI was associated with a lower risk of DKD among people aged <60 years [HR = 0.21; 95% CI: 0.04–0.96; *p* = 0.044]. **Conclusions:** A ≥5% increase in BMI in three years may be a risk factor for new-onset or progressive DKD among people with T2DM and normal BMI. Conversely, a ≥5% decrease in BMI may be associated with renal protection in non-elderly individuals within the population.

## 1. Introduction

In recent decades, the global prevalence of diabetes has sharply increased. The number of affected adults will reach 853 million by 2025, with type 2 diabetes mellitus (T2DM) being the predominant form [1]. Diabetic kidney disease (DKD), an important microvascular complication of T2DM, is characterized by persistent proteinuria and/or a decline in estimated glomerular filtration rate (eGFR). It is not only the leading cause of end-stage kidney disease (ESKD) [2,3] but it also substantially increases the risk of cardiovascular events and all-cause mortality [2,4].

Obesity is a well-established risk factor for T2DM, and the majority of people with T2DM are overweight or obese. Numerous studies have demonstrated that obesity accelerates the development of DKD [5,6,7,8]. Data from the ACCORD trial indicate that greater weight variability in obese people with T2DM is associated with a higher risk of DKD [9]. Furthermore, the risk of DKD is significantly higher in people with class I–III obesity compared with those of normal body mass index (BMI) [10]. Weight reduction in people with T2DM has been shown to improve multiple cardiovascular disease (CVD) risk factors [11,12]; moreover, both excessive weight gain and weight loss have been linked to adverse outcomes. For example, weight fluctuations have been associated with an increased risk of myocardial infarction, stroke, and all-cause mortality in T2DM [4]. A recent study involving 6040 participants reported that high BMI variability is associated with increased risk of DKD [13].

However, most studies have focused on overweight or obese individuals with T2DM and explored the association between weight changes and CVD, chronic kidney disease (CKD), and ESKD. It remains unclear whether a similar pattern exists in normal-weight people with T2DM. Notably, a recent study revealed that a BMI below 18.5 kg/m^2^ is an independent risk factor for ESKD, and individuals with BMI changes within ±5% had the lowest risk, whereas weight loss or gain exceeding 10% markedly increased risk [14].

Based on data from the Chongqing Diabetes Registry (CDR) cohort, this study aimed to investigate the association between three-year BMI changes and the risk of new-onset or progressive DKD in people with T2DM with a normal BMI.

## 2. Method

### 2.1. Data Collection and Laboratory Assays

This cohort study was approved by the Ethics Committee of the First Affiliated Hospital of Chongqing Medical University in China. All participants from the CDR database provided written informed consent. Data from people with T2DM were collected. Demographic information and clinical risk factors were extracted from the CDR at recruitment and during follow-up, including age, sex, smoking status, alcohol use, blood pressure, glycated hemoglobin (HbA1c), total cholesterol (TC), triglyceride (TG), high-density lipoprotein cholesterol (HDL-C), low-density lipoprotein cholesterol (LDL-C), the urine albumin to creatinine ratio (UACR), and eGFR, which was calculated using the Chronic Kidney Disease Epidemiology Collaboration (CKD-EPI) equation. Biological samples of the cohort population were collected and assayed in the laboratory. The change rate of the body mass index (BMI) was calculated as follows: (BMI at third year − baseline BMI)/baseline BMI × 100%. The average follow-up time for CDR participants was 3.4 years. The eGFR change was calculated as follows: (eGFR at third year − baseline eGFR). Due to the non-normal distribution of UACR, the UACR change was calculated as follows: (log_10_(UACR at third year) − log_10_(baseline UACR)).

### 2.2. Study Population

**Figure 1:**
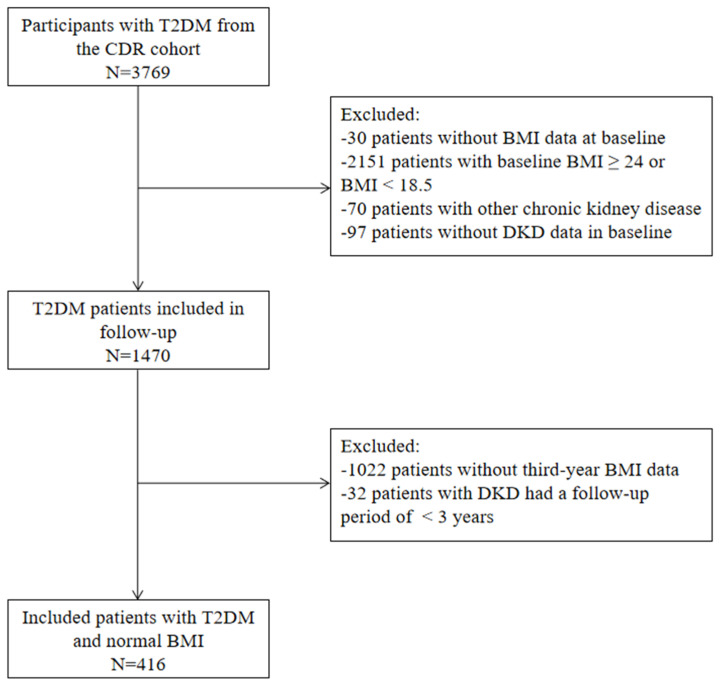
Flow chart of study population. Abbreviations: CDR, Chongqing Diabetes Registry; T2DM, type 2 diabetes mellitus; BMI, body mass index.

The CDR is a prospective cohort study that mainly observes the occurrence and development of diabetic complications and their related risk factors. Since May 2018, people with type 2 diabetes who are willing to participate in the follow-up have been recruited from the inpatient and outpatient departments of the First Affiliated Hospital of Chongqing Medical University. Most participants came from Chongqing. Their DKD events were followed up in the First Affiliated Hospital of Chongqing Medical University. To date, 3769 people have been included in the study. Following enrollment, people are followed up on site after 1, 3, and 6 years, as well as by phone every half year. Due to disruptions caused by the COVID-19 outbreak at the start of 2019, a significant number of people were unable to complete their scheduled third-year on-site visit. Participants were excluded if they had a baseline BMI ≥ 24 kg/m^2^ or <18.5 kg/m^2^, a diagnosis of non-diabetic kidney disease, or lacked third-year data regarding BMI or UACR/eGFR. Ultimately, 416 people with T2DM completed the medical examinations and provided full follow-up data; linear regression was used to analyze the relationship between BMI change and the eGFR or UACR change in the three years, and Cox proportional hazards regression analysis was performed to assess the association between BMI change and new-onset or progressive DKD (Figure 1).

### 2.3. Definition of New-Onset and Progressive DKD

In people with T2DM without other types of kidney disease, new-onset DKD was defined as having an eGFR from ≥60 mL/min/1.73 m^2^ to <60 mL/min/1.73 m^2^ or/and UACR from normoalbuminuria (UACR < 30 mg/g) to UACR ≥ 30 mg/g. The definition of DKD progression is as follows: for DKD people, a ≥40% eGFR decline or incidence of ESKD/renal function lost, or the UACR (mg/g) increases from <30 to 30–300 or 30–300 to ≥300 [15].

### 2.4. Statistical Analysis

People with T2DM were categorized into three groups based on the BMI change rate over three years: stable BMI (<5% change), decreased BMI (≥5% decrease), and increased BMI (≥5% increase). The BMI change groups were defined based on a previous weight change study [14,16]. If the distribution of a continuous variable is approximately normal, the baseline characteristics are shown as the mean and standard deviation (SD), with intergroup differences assessed via one-way analysis. If a skewed distribution is observed, the data are shown as the median and interquartile range (IQR). Categorical variables are summarized as numbers (percentages) with intergroup differences assessed using the χ^2^ test. Linear regression models were employed to estimate β, and standard β was used to evaluate the association between BMI change and the eGFR or UACR change over the three years. Cox proportional hazards regression models were employed to estimate hazard ratios (HRs) and 95% confidence intervals (CIs) to evaluate the independent association between BMI change groups and DKD incidence. The models were adjusted as follows: Model 1, unadjusted; Model 2, adjusted for age and sex; Model 3, further adjusted for smoking status, systolic blood pressure (SBP), HbA1c, TC, triglycerides (TG), HDL-C, LDL-C, diabetes duration, intensity of physical activity, tumor, frailty, coronary heart disease, stroke/TIA, heart failure, waist–hip ratio, and protective medications for diabetic kidney disease duration, including GLP-1, SGLT-2i, and ACEI/ARB. Covariates were selected based on the established DKD risk factors. Cox regression analyses were used again to present the results by gender (male/female), age (≥60years/<60years), and HbA1c (≥7%/<7%) to determine whether the association of BMI change rate with incident DKD was different across subgroups. The results are shown as forest plots. All statistical analyses were conducted using IBM SPSS Statistics version 26 and R studio 4.52. A two-sided *p*-value < 0.05 was considered statistically significant.

## 3. Results

### 3.1. Baseline Characteristics

Table 1 shows the baseline characteristics of the study population based on different BMI change groups (*n* = 416). Among the 416 CDR participants with T2DM, the average age was 58.66 years, with 208 males and 208 females. During the average follow-up period of 3.4 years, there were 96 new cases of new-onset DKD (23.1%) and 41 new cases of progressive DKD (9.9%).

Based on the linear regression results, BMI change showed a negative correlation with eGFR change and a positive correlation with UACR change over the three years (Supplementary Table S1).

### 3.2. Cox Regression for New-Onset or Progressive DKD According to the BMI Change Rate

The association between the three-year BMI change rates and new-onset or progressive DKD was evaluated using Cox proportional hazards models with 416 people with T2DM. The results show that the increased BMI group (≥5% increase) had a higher risk of new-onset or progressive DKD (HR = 1.67, 95% CI: 1.15–2.43, *p* = 0.007), taking the stable group (<5% change) as the reference. It showed a similar result when BMI change was used as a continuous variable (HR = 1.05, 95% CI: 1.02–1.07, *p* < 0.001).

After adjusting for age and gender in Model 2, the association remained stable (HR = 1.73, 95% CI: 1.19–2.51, *p* = 0.004). After further adjustment for smoking, SBP, HbA1c, TC, TG, HDL-C, LDL-C, diabetes duration, intensity of physical activity, tumor, frailty, coronary heart disease, stroke/TIA, heart failure, waist–hip ratio, and protective medications for diabetic kidney disease duration in Model 3, the association remained significant (HR = 1.79, 95% CI: 1.04–3.07, *p* = 0.036). On the contrary, the decreased BMI group (≥5% decrease) did not show a significant correlation with the risk of new-onset or progressive DKD in all models (*p* > 0.05). The result was similar when BMI change was a continuous variable (HR = 1.05, 95% CI: 1.02–1.09, *p* = 0.001) (Table 2).

**Figure 2:**
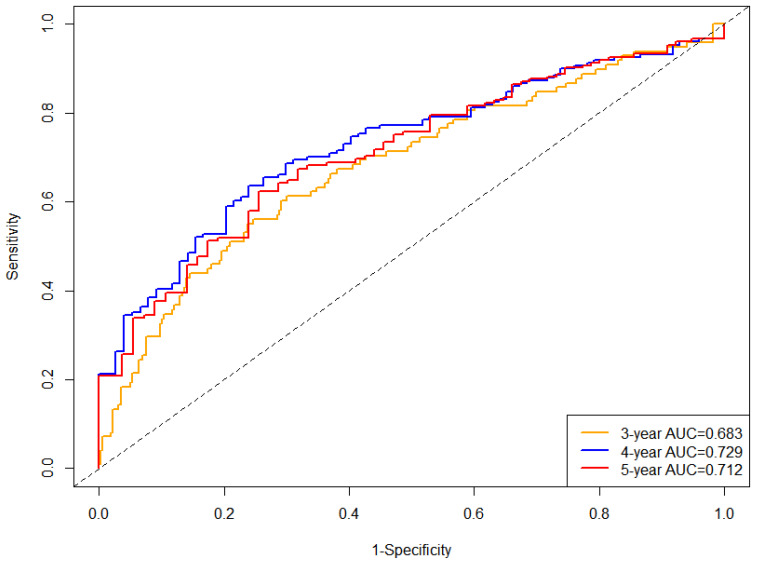
Time-dependent receiver operating characteristic analysis of three-year BMI change rate in predicting new-onset or progressive DKD. Time-dependent receiver operating characteristic analysis of BMI changes and new-onset or progressive DKD in the CDR cohort after adjusting for the following variables in Model 3: age, gender, smoking, SBP, HbA1c, TC, TG, HDL-C, LDL-C, diabetes duration, intensity of physical activity, tumor, frailty, coronary heart disease, stroke/TIA, heart failure, waist–hip ratio, and protective medications for diabetic kidney disease duration. Abbreviations: BMI, body mass index; SBP, systolic blood pressure; HbA1c, glycated hemoglobin; TC, total cholesterol; TG, triglyceride; HDL-C, high-density lipoprotein cholesterol; LDL-C, low-density lipoprotein cholesterol; TIA, transient ischemic attack.

The time-dependent ROC curve also shows that Model 3 performs well in predicting new-onset or progressive DKD (AUC: 3-year 0.683; 4-year 0.729; 5-year 0.712) (Figure 2).

### 3.3. Association of the BMI Change Rate with New-Onset or Progressive DKD in Subgroup Analysis

**Figure 3:**
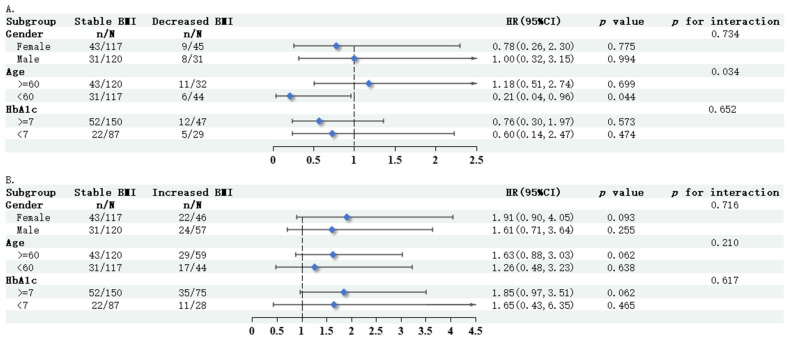
Hazard ratios for Cox regression in Model 3 on the association between decreased BMI (**A**) or increased BMI (**B**) and new-onset or progressive DKD in different subgroups. Cox regression analysis of BMI changes and new-onset or progressive DKD in the CDR cohort after adjusting for the following variables in Model 3: age, gender, smoking, SBP, HbA1c, TC, TG, HDL-C, LDL-C, diabetes duration, intensity of physical activity, tumor, frailty, coronary heart disease, stroke/TIA, heart failure, waist–hip ratio, and protective medications for diabetic kidney disease duration. Abbreviations: DKD, diabetic kidney disease; HbA1c, glycated hemoglobin; TIA, transient ischemic attack.

To confirm the stability of the results, the correlation between BMI change rate and new-onset or progressive DKD was investigated across subgroups based on gender, age, and HbA1c. As shown in Figure 3, we performed analyses after adjusting for age, gender, smoking, SBP, HbA1c, TC, TG, HDL-C, LDL-C, diabetes duration, intensity of physical activity, tumor, frailty, coronary heart disease, stroke/TIA, heart failure, waist–hip ratio, and protective medications for diabetic kidney disease duration. In the subgroup with non-elderly people (<60 years), multivariate Cox regression showed that individuals with decreased BMI had a lower risk of new-onset or progressive DKD compared to those with stable BMI (HR = 0.21, 95% CI = 0.04–0.96, *p* = 0.044 vs. HR = 1.17, 95% CI = 0.51–2.74, *p* = 0.699), as well as a significant interaction (*p* for interaction = 0.034). No significant interactions were observed in the other subgroups (*p* for interaction > 0.05).

## 4. Discussion

This study shows that an increased BMI over three years is associated with a significantly higher risk of new-onset or progressive DKD among people with type 2 diabetes and a normal baseline BMI. It may be a risk factor for DKD. Conversely, a decrease in BMI is associated with a lower risk of DKD in younger and middle-aged individuals. Furthermore, the baseline showed that the people with increased BMI had higher HbA1c levels. The result remains stable after adjusting for HbA1c. These findings suggest that longitudinal BMI change, rather than baseline BMI alone, may play an important role in identifying individuals at increased risk of DKD within the normal-weight diabetic population. Therefore, the three-year BMI change rate might be a useful clinical indicator to guide individualized long-term weight management strategies for people with type 2 diabetes.

Previous studies have shown that the overweight and obese population has an increased risk of DKD [17]. Moreover, diabetes guidelines emphasize the importance of long-term weight management [18]. However, it remains uncertain whether people with T2DM with normal weight should intervene in their weight changes to reduce new-onset or progressive DKD in clinical practice. Previous studies have emphasized the correlation between BMI and DKD [7,8,19]. However, studies focusing on the association between weight changes and new-onset or progressive DKD are lacking. A cohort study explored how high weight variability is associated with an increased risk of new-onset DKD in people with T2DM [13]. Due to the complexity of its calculation, this indicator is not widely used in clinical practice. Note that the study did not distinguish between weight loss and weight gain. It remains unclear whether weight loss is beneficial to DKD; different studies have reported different results. A longitudinal study showed that weight loss delayed the decline in eGFR [20]. However, another study showed that weight loss >10% was associated with renal function decline [14]. In addition, underweight was associated with an increased risk of CKD [21]. Unfortunately, these studies did not analyze the association between weight changes and DKD.

The underlying mechanism of the association between weight changes and DKD remains incompletely understood. However, weight changes affect a number of indicators, such as blood sugar levels, blood pressure, blood lipid levels, and the level of urinary albumin [12]. Based on a previous study, some potential mechanisms were proposed. First, weight changes may affect systemic or local inflammation. Some studies have revealed a connection between body weight and chronic inflammation. For example, IL-6 is an inflammatory factor, and the relationship between weight gain and increased IL-6 levels has been confirmed [22]. Furthermore, high IL-6 levels contribute to fibrotic and ET-1 gene expression in the kidney [23]. Thus, long-term chronic inflammation may lead to DKD progression [24]. On the contrary, MCP-1 levels are significantly decreased after weight loss [25]. MCP-1 induces the accumulation of macrophages and renal inflammatory response [26]. Therefore, low MCP-1 levels might suggest improvement in inflammatory levels in patients. Second, high blood pressure is very common among people with T2DM. Hypertension can result in damage to renal blood vessels, podocyte detachment, and epithelial-to-mesenchymal transition, thereby disrupting the negative charge barrier. Ultimately, hypertension causes proteinuria and kidney injury [27]. A study has shown that hypertension increased the risk of developing DKD [28]. In addition, a weight loss of 5% to <10% will lead to a decrease in systolic and diastolic blood pressure [12]. Third, it is well known that blood lipid level plays an indispensable role in DKD. As a significant risk factor for DKD, the connection between blood lipids and DKD has been confirmed [29]. Finally, the cumulative incidence of ESRD has been observed to be 40% in people with diabetes mellitus after 10 years of onset of albuminuria [30]. However, the mechanism by which albuminuria affects DKD remains unclear. Meanwhile, some diseases also cause weight changes through fluid retention, such as incipient kidney disease or heart failure. Therefore, it is worth investigating whether changes in fat mass and lean mass are better predictors of DKD than BMI. Weight loss benefits were found in the elderly. This may be because weight loss in some elderly individuals is due to an underlying cachexic condition or poor blood glucose control, such as in cancer, sarcopenia, or infection [31]. These age-related adverse factors may mask the renal benefits from weight loss. Therefore, it remains uncertain whether intentional weight loss confers renal benefits in the elderly. Nonetheless, we believe that intentional weight loss may still hold promise for renal benefits in the elderly with normal weight. Meanwhile, doctors need to pay attention to elderly people with decreased BMI and determine the reasons for the weight loss, especially weight loss caused by disease.

This study has several strengths. To our knowledge, this is the first study to evaluate the association between BMI change and the risk of new-onset or progressive DKD in people with T2DM and normal weight. Secondly, this study reveals that people with increased BMI have a higher risk of new-onset or progressive DKD. Finally, the results from young and middle-aged people with T2DM highlight the renal benefits associated with decreased BMI.

Several limitations should be acknowledged in this study. First, most participants were recruited from China, and the generalizability of these findings to other ethnicities or regions remains uncertain. Second, the sample size was relatively small, and larger cohort studies are warranted to verify the robustness of the observed association between BMI change and the risk of new-onset or progressive DKD. Third, the follow-up duration was relatively short; most people were followed up for three years, which may have limited the ability to fully capture the long-term association between BMI change and DKD progression. Due to these limitations, the distinction between intentional and unintentional weight loss may have been insufficiently robust in this study. Stronger evidence is expected to demonstrate the differences in weight changes among different age groups on DKD in the future, as well as to clarify the effect of intentional and unintentional weight loss.

## 5. Conclusions

An increased three-year BMI change rate is associated with an increased risk of new-onset or progressive DKD. Thus, it may serve as an independent predictive indicator for new-onset or progressive DKD in people with T2DM. In addition, a decreased three-year BMI change rate may indicate a protective effect for non-elderly people with normal weight and T2DM.

## Figures and Tables

**Table 1:** Baseline characteristics of participants from the CDR cohort by BMI categories.

	Stable BMI	Decreased BMI	Increased BMI	*p*-Value
	*n* = 237	*n* = 76	*n* = 103	
BMI change (%)	−0.07	−8.22	11.05	
Age (years)	58.85 ± 9.97	57.64 ± 9.80	58.97 ± 11.30	0.632
Male (*n* (%))	120 (50.63%)	31 (40.79%)	57 (55.34%)	0.150
BMI (kg/m^2^)	22.05 ± 1.39	22.19 ± 1.31	21.71 ± 1.49	0.045
SBP (mm/Hg)	128.85 ± 16.13	127.05 ± 17.94	130.40 ± 16.32	0.408
Smoking (*n* (%))	8786 (36.29%)	18 (23.68%)	44 (42.72%)	0.030
Alcohol (*n* (%))	54 (22.78%)	10 (13.16%)	24 (23.30%)	0.167
FPG (mmol/L)	8.13 ± 2.80	8.54 ± 3.92	8.81 ± 3.57	0.202
HbA1c (%)	8.22 ± 2.17	8.10 ± 1.92	9.18 ± 2.71	0.003
TC (mmol/L)	4.36 ± 1.10	4.70 ± 1.38	4.32 ± 1.05	0.049
TG (mmol/L)	1.30 (0.93~1.98)	1.23 (0.95~1.86)	1.20 (0.87~1.94)	0.478
HDL-C (mmol/L)	1.27 ± 0.40	1.39 ± 0.42	1.21 ± 0.39	0.011
LDL-C (mmol/L)	2.63 ± 1.07	2.80 ± 1.08	2.53 ± 0.90	0.223
eGFR (ml/min/1.73 m^2^)	86.02 ± 20.90	89.48 ± 20.27	86.41 ± 21.45	0.452
UACR (mg/g)	8.2 (3.9~20.2)	9.9 (4.0~27.1)	8.7 (4.3~23.8)	0.344
Protective medication for DKD (*n* (%))	60 (25.32%)	14 (18.42%)	33 (32.04%)	0.117
Diabetes duration (years)	9.42 (4.08~14.58)	10.00 (3.94~14.92)	8.25 (1.83~14.92)	0.233
Physical activity (*n* (%))	174 (73.42%)	58 (76.32%)	66 (64.08%)	0.130
Tumor (*n* (%))	20 (8.44%)	7 (9.21%)	13 (12.62%)	0.481
Frailty (*n* (%))	69 (29.11%)	31 (40.79%)	34 (33.01%)	0.163
Coronary heart disease (*n* (%))	17 (7.17%)	7 (9.21%)	8 (7.77%)	0.845
Stroke/TIA (*n* (%))	9 (3.80%)	9 (11.84%)	3 (2.91%)	0.011
Heart failure (*n* (%))	1 (0.42%)	2 (2.63%)	1 (0.97%)	0.229

Notes: Data are expressed as mean (SD), percentages, or medians [IQR]. Abbreviations: BMI, body mass index; SBP, systolic blood pressure; FPG, fasting plasma glucose; HbA1c, glycated hemoglobin; TC, total cholesterol; TG, triglyceride; HDL-C, high-density lipoprotein cholesterol; LDL-C, low-density lipoprotein cholesterol; eGFR, estimated glomerular filtration rate; UACR, urinary albumin-to-creatinine ratio; TIA, transient ischemic attack.

**Table 2:** Cox proportional hazards regression analysis of association between three-year BMI change rate and risk of new-onset or progressive DKD.

Model	BMI Change (Per 1% Increased)		Stable BMI	Decreased BMI		Increased BMI	
	HR (95%CI)	*p*-Value	Reference	HR (95%CI)	*p*-Value	HR (95%CI)	*p*-Value
Model 1	1.05 (1.02, 1.07)	<0.001	1	0.64 (0.38, 1.09)	0.1	1.67 (1.15, 2.43)	0.007
Model 2	1.05 (1.02, 1.07)	<0.001	1	0.66 (0.39, 1.13)	0.128	1.73 (1.19, 2.51)	0.004
Model 3	1.05 (1.02, 1.09)	0.001	1	0.74 (0.35, 1.54)	0.416	1.79 (1.04, 3.07)	0.036

Model 1 was unadjusted. Model 2 was adjusted for age and gender. Model 3 was further adjusted for smoking, SBP, HbA1c, TC, TG, HDL-C, LDL-C, diabetes duration, intensity of physical activity, tumor, frailty, coronary heart disease, stroke/TIA, heart failure, waist–hip ratio, and protective medications for diabetic kidney disease duration. Abbreviations: BMI, body mass index.

## Data Availability

The data supporting the study results are available from the corresponding author upon reasonable request.

## Supplementary Materials

The following supporting information can be downloaded at: https://www.mdpi.com/article/10.3390/jcm15083125/s1. Table S1: Associations Between Renal 3-year BMI change rat and eGFR change or UACR change Based on Linear Regression Models.
