# Supplementary material for: Association of BMI Change with New-Onset or Progressive Diabetic Kidney Disease in People with Normal-Weight Type 2 Diabetes
Source: J Clin Med. 2026 Apr 20;15(8):3125. doi: 10.3390/jcm15083125 (PMC13117657; doi:10.3390/jcm15083125)
Supplement: Supplementary file 1 [file jcm-15-03125-s001.zip › jcm-4202856-supplementary.pdf]

Supplementary Table S1: Associations Between Renal 3-year BMI change rat and eGFR change or UACR change Based on Linear Regression Models.

	ΔeGFR		$\Delta \log_{10}$UACR	
	Std β	*p*-value	Std β	*p*-value
model1	-0.124	0.016	0.155	0.003
model2	-0.150	0.002	0.153	0.004
model3	-0.141	0.004	0.128	0.018

Model 1 was unadjusted. Model 2 was adjusted for age and gender. Model 3 was further adjusted for smoking, SBP, HbA1c, TC, TG, HDL-C, LDL-C, diabetic kidney disease protective medication.

Abbreviations: BMI, Body Mass Index; SBP, Systolic Blood Pressure; HbA1c, Glycated Hemoglobin; TC, Total Cholesterol; TG,Triglyceride; HDL-C, High-density Lipoprotein Cholesterol; LDL-C, Low-density Lipoprotein Cholesterol.
